# Total proteome turbidity assay for tracking global protein aggregation in the natural cellular environment

**DOI:** 10.14440/jbm.2017.148

**Published:** 2017-04-25

**Authors:** Merav D. Shmueli, Noa Hizkiahou, Sivan Peled, Ehud Gazit, Daniel Segal

**Affiliations:** Department of Molecular Microbiology and Biotechnology and the Interdisciplinary Sagol School of Neurosciences, George S. Wise Faculty of Life Sciences, Aviv University, Aviv 69978, Israel

**Keywords:** turbidity, protein aggregation, cancer, Alzheimer’s disease, VHL, ALS, Parkinson's disease

## Abstract

Proteome homeostasis is crucial for optimal cellular function and survival in the face of various stressful impacts. This entails preservation of a balance between protein synthesis, folding, degradation, and trafficking collectively termed proteostasis. A hallmark of proteostasis failure, which underlies various diseases, is enhanced misfolding and aggregation of proteins. Here we adapted the measurement of protein turbidity, which is commonly used to evaluate aggregation of single purified proteins, for monitoring propensity for aggregation of the entire soluble cellular proteome incubated *in vitro* for several hours. We show that over-expression of an aggregation-prone protein or applying endoplasmic-reticulum (ER) stress to either cells in culture or to the intact organism, Drosophila, enhances the rise in turbidity of the global soluble proteome compared to untreated cells. Additionally, given that Alzheimer’s disease (AD) is known to involve ER stress and aggregation of proteins, we demonstrate that the soluble fraction of brain extracts from AD patients displays markedly higher rise of global proteome turbidity than in healthy counterparts. This assay could be valuable for various biological, medical and biotechnological applications.

## INTRODUCTION

Protein aggregation is associated with various diseases of unrelated etiologies. It accompanies various extracellular and intracellular stresses, such as endoplasmic-reticulum (ER) stress, enhanced unfolded protein response (UPR) activity, proteasome malfunction, heat shock and oxidative stress [1-3]. An increasing number of proteins are identified as intrinsically disordered even in their native conformation, making them especially prone to aggregation [4]. Typical examples include Amyloid-β (Aβ) and tau, involved in Alzheimer’s disease (AD), α-synuclein (α-syn) the main agent in Parkinson’s disease (PD) and PrP in prion diseases [5]. Aggregates of tumor suppressor proteins such as p53 and pVHL have also been documented and were related to the process of transformation [6,7].

A protein may become prone to aggregation due to various characteristics of its structure: its native structure may be metastable (*i.e.* intrinsically disordered) [8], missense mutations may alter its conformation and partial degradation may result in aggregative fragments [9,10]. The aggregation process begins with the formation of aggregation nuclei, whose growth is the rate-limiting step of this process [11]. Once a nucleation seed is formed, aggregate growth is thought to proceed rapidly by further association of either monomers or oligomers to the formed nucleus [12]. A protein may aggregate along various pathways depending on the environmental conditions and types of cellular stress [13]. Usually, misfolding and consequent aggregation of a protein leads to loss of functionality. In addition, the aggregates themselves may be cytotoxic [14]. Importantly, increasing evidence suggests that the aggregating protein could affect other proteins leading to loss of their function. For example, misfolded prion (PrP^Sc^) recruits, and corrupts normal PrP^c^ [15]; mutant p53 was shown to co-aggregate with its paralogs p63 and p73 [16]; oligomers of phosphorylated Tau were found to co-aggregate with α-syn revealing molecular cross-talk between different aggregation pathways involved in neurodegeneration [17]. Thus, aggregation of a single protein species can lead to aggregation cascade involving additional proteins, resulting in broad impact on the cell.

A complex cellular machinery for ensuring proteostasis has evolved to handle misfolding and consequent aggregation of proteins. It commences with the recognition of the misfolded protein, and the attempt to restore its normal folding (*e.g.*, by molecular chaperones). If not successful the UPR is activated together with stress-responsive signaling aimed at targeting the protein for either degradation, sequestration in inclusion bodies, or activation of apoptosis [1,18,19].

Overloading the machinery involved in maintaining proteostasis with a specific misfolded protein can, indirectly, lead to its inability to handle aggregation of other proteins, especially those that are intrinsically unstable and prone to aggregation (*e.g.*, p53) [20].

Current approaches for monitoring protein aggregation rely on isolating the specific protein of interest. They include use of conformational sensitive dyes (*e.g.*, Thioflavin-S/T), direct spectroscopy (*e.g.*, FTIR, NMR, TEM and SEM), light scattering (*e.g.*, turbidity, CD and DLS or SLS). In addition, aggregation sensitive reporters (*e.g.*, FRET), native and denatured acryl amide gels have been employed. Indirect methods for evaluating protein aggregation can also be used such as monitoring the activation level of chaperones, or relying on various readouts of the activation of the UPR machinery, ER-stress and proteasomal degradation [21].

Here we present a facile method for monitoring global propensity for aggregation of proteins in the cell, using turbidity assay of the soluble fraction of total protein extracts incubated *in vitro* for several hours. Employing protein extraction protocols commonly used for each of the following we show that over-expression of a specific protein or applying ER stress to either cells in culture or to the intact organism, Drosophila, enhances the rise in proteome turbidity compared to untreated cells. Given that AD is known to involve aggregation of amyloidogenic polypeptides and enhanced ER stress [22,23], we demonstrate that soluble fractions of brain cells from postmortem AD patients display markedly higher rise of proteome turbidity than extracts from healthy counterparts. This assay could be valuable for a broad range of applications in biomedicine and biotechnology.

## METHODS

### Drosophila strains and genetics

The following fly strains were used: Oregon R (OR) served as wild-type flies. The control flies expressing only the relevant Gal4 driver were obtained by crossing OR flies with the flies carrying the appropriate GAL4 driver (**Table 1**).

Tau-expressing and Aβ42-expressing flies shown in **Table 1** were generated from lines # 51362 (w [1118]; P{w [+mC] = UAS-Tau.wt}1.13) and # 33769 (w [1118]; P{w [+mC] = UAS-APP.Abeta42.B}m26a), respectively (Bloomington Drosophila stock center, Indiana University).

Wild type human pVHL and its N78S, F136L and Y98H mutant versions were cloned into the pUASTattB vector by standard procedures [6] using EcoRI and XhoI restriction enzymes. These expression vectors were used to transform the phiC31-integrase-mediated recombination flies [25] (BestGene, CA). We used transgenic strains carrying stable insertions on chromosome 3. Tubulin-Gal4 was kindly provided by O. Gerlitz (Hebrew University of Jerusalem, Israel), and was used for inducing UAS-dependent ubiquitous expression.

All strains were reared on standard cornmeal-molasses medium at 25°C. Experiments were carried out at 25°C. Adult offspring (F1) from the crosses were collected up to 9 days after the beginning of their eclosion at 25°C in order to avoid offspring from the next generation (F2).

### Cell culture

SH-SY5Y (human neuroblastoma) cells were obtained from the American Type Culture Collection (ATCC) and cultured in DMEM F12 1:1 containing 10% fetal bovine serum, 2 mM L-glutamine, 1000 U/ml penicillin-G, 1 mg/ml streptomycin sulfate and 1 mM sodium pyruvate at 37°C under 5% CO_2_ (v/v). To generate SH-SY5Y cells stably expressing HA-tagged proteins, we used a CMV retroviral system. The infected cells were selected using puromycin.

### Human brain tissue

Frozen autopsied frontal cerebral cortices, five AD and five controls were kindly provided by the University of Manchester Brain Bank. The age, gender and Braak stages of these samples are listed in **Table 2**. The number of samples per group was *N* = 5.

The use of the human brain tissue was in accordance with all relevant Codes of Practice of the Human Tissue Authority (HTA) and approved by the institutional review board of Aviv University and of the University of Manchester Brain Bank.

### Protein extraction

Drosophila: Collected whole flies or fly heads were homogenized in 1:1 volume of extraction buffer [5 mM MgCl_2_, 1 mM DTT, 1 mM PMSF, 1 × Complete protease inhibitor cocktail (Roche), 1 × phosphate buffer saline (PBS), pH 7.5]. The samples were centrifuged for 30 min at 20000 g at 4°C for removal of non-soluble particles and the resulting supernatants were used for further analyses [26].

Cell culture: cells were grown to 80%–100% confluence in 100 mm culture plates. The plates were rinsed with cold PBS and cells were extracted in buffer (5 mM MgCl_2_, 1 mM DTT, 1 mM PMSF, 1 × Complete protease inhibitor cocktail (Roche), 1 × PBS, pH 7.5) followed by three cycles of sonication on ice. Lysed cells were centrifuged for 15 min at 20000 g at 4°C for removal of non-soluble particles and the resulting supernatants were used for further analyses. Protein extraction from cell culture was adapted from [27,28].

Brain tissue: was homogenized using BULLET BLENDER^®^ (Next Advance, Inc.) after several washes with PBS in ice-cold homogenization buffer, consisting of 50 mM Tris–HCl, pH 7.4, 8.5% sucrose, 2.0 mM EDTA, 100 mM GlcNAc and Complete protease inhibitor cocktail (Roche). The homogenates were centrifuged at 20000 g for 20 min at 4°C for removal of non-soluble particles and the resulting supernatants were used for further analyses. Protein extraction from cell culture was adapted from [29,30].

Protein concentration was measured using Bradford or BCA reagents (Sigma-Aldrich).

### Turbidity measurements

Supernatants were diluted in relevant extraction buffer to a desired concentration 1 mg/ml. Appropriate number of biological (*n* > 3) and technical (*n* > 3) replicates were conducted for each experiment. For choosing the wavelength at which turbidity will be measured we examined the absorbance spectra of total soluble protein extracts and observed no significant peak at 340 nm (**Fig. S1**). Therefore turbidity was monitored at 340 nm, using a Synergy HTX plate reader (Biotek) and polypropylene 96-well plates (Corning) in 100 μl volume. Plates were shaken gently for 5 s prior to each measurement cycle and incubated at 37°C. All optical densities were measured at 340 nm wavelength and their first measurement (O.D.(T_0_)) was subtracted in order to eliminate background absorbance of the samples.

### Quantitative periodic acid Schiff staining

Prior to experiment, samples were adjusted to identical protein concentrations (measurement was performed by BCA reagent, PIR-2325, Pierce). To 25 µl protein lysate solution (2 mg/ml) in each well of a 96 well plate, 120 µl of 0.06% periodic acid solution (375810, Sigma-Aldrich) in freshly made 7% acetic acid were added. The microtiter plate was covered with a plastic seal and incubated at 37°C for 1.5 h without shaking. The plate was allowed to cool to room temperature before 100 µl of Schiff’s reagent were added to each well (395-2, Sigma-Aldrich). The microtiter plate was covered again with a plastic seal and was shaken for 5 min. Color was allowed to develop at room temperature for additional 30 min before absorbance was recorded at 550 nm.

### 8-anilino-1-naphthalene sulfonic acid fluorescence studies

Fluorescence emission at 460/40 nm was generated with excitation wavelength of 350 nm using a Synergy HTX plate reader (Biotek) and polypropylene 96-well plates (Corning) in 100 μl volume. The first solution contained 10 µM 8-anilino-1-naphthalene sulfonic acid (ANS) in PBS buffer. The other solutions were similar and contained total soluble protein extract at a final concentration of 1 mg/ml. Baseline corrections were made with buffer lacking proteins and ANS. Appropriate number of biological (*n* > 3) and technical (*n* > 3) replicates were conducted for each experiment. Plates were shaken gently for 5 s prior to each measurement cycle and incubated at 37°C.

### Immunoblotting

Laemmli sample buffer (BioRad) was added to equal amount of total protein extract from each sample, followed by boiling for 10 min, centrifugation for 5 min at 14000 RPM and analysis by SDS-PAGE and Western blot. Protein extracts were resolved on 4%–20% gradient SDS-PAGE (GeBA, Israel) and transferred on to PVDF membrane using iBlot 7-Minute Blotting System (Life Technologies). The membrane was blocked for 1 h in blocking solution (5% milk powder, 0.02% sodium-azide in 1 × TBS), and then incubated with the primary antibody diluted in blocking solution. The membrane was then washed 3 times for 15 min each in TTBS (0.1% Tween-20 in 1 × TBS), incubated for 1 h with the secondary antibody and washed 3 times for 10 min in TTBS. The membrane was developed using EZ-ECL (Biological Industries, Israel), according to the manufacturer’s instructions, and exposed to Fuji Medical X-Ray Film for up to 5 min. Films were developed using Kodak X-OMAT 2000.

Antibodies used: anti β actin 1:5000 (ab8224, abcam), anti Tau protein 1:1000 (5A6, Hybridoma bank), anti Aβ 1:500 (6E10, Biolegend), anti pVHL 1:1000 (VHL40, Santa Cruz Biotechnology, Inc.) anti-HA 1:1000 (sc7392 Santa Cruz Biotechnology, Inc.) anti-TDP43 1:1000 (AP-10782-2 Proteintech).

Secondary antibodies used were from Santa Cruz Biotechnology, Inc. (horseradish peroxidase-coupled goat anti-mouse and goat anti-rabbit antibodies).

### Reverse transcription and real-time PCR

Total RNA from 10 adult flies was isolated with RNeasy mini kit with on-column DNase digestion (Qiagen). First-strand cDNA was generated by using the Verso cDNA Kit (Thermo). Real-time polymerase chain reaction (PCR) was performed in triplicate with KAPA Fast SYBR master mix (KAPA Biosystems) and the StepOnePlus™ Real-Time PCR Systems (Life Technologies, Israel). Primers for rp49, pVHL were purchased from HyLabs (Israel). All values were normalized to the level of rp49 mRNA abundance and to the wild type (Oregon R) flies. Each primers pair was calibrated using the Absolute Quantification program with increasing concentrations of cDNA, from 1:32 to 1:1 dilutions of the original cDNA. Primers used were: rp49 (forward 5’- ACC GAT GTT GGG CAT CAG ATA-3’ ; reverse 5’- TAA GCT GTC GCA CAA ATG GC-3’) and pVHL (forward 5’ CCT CCC AGG TCA TCT GCA AT-3’; reverse 5’- GTT AAC CAG AAG CCC ATC GTG TG-3’).

### Compound preparation, concentration and treatment

Stock solutions of tunicamycin or thapsigargin (Sigma-Aldrich) were prepared in dimethylsulfoxide. For tunicamycin treatment, flies were grown, in the dark, for their entire life time on cornmeal-molasses medium containing 10 μM tunicamycin. They were collected 7 d after eclosion from the pupal case, cells were exposed to 10 μM tunicamycin for 24 h. For thapsigargin treatment, cells were exposed to 10 μM for 24 h. The control cells or flies received the same amount of solvents.

### Statistics

All experiments were repeated three times or more and the significant difference was calculated using a student *t*-test and ANOVA for evaluating statistical significance of the observed differences. *P < 0.05, **P < 0.01, ***P < 0.001.

## RESULTS AND DISCUSSION

### Induced ER stress results in elevated global proteome turbidity in *Drosophila* model

**Figure 1.**
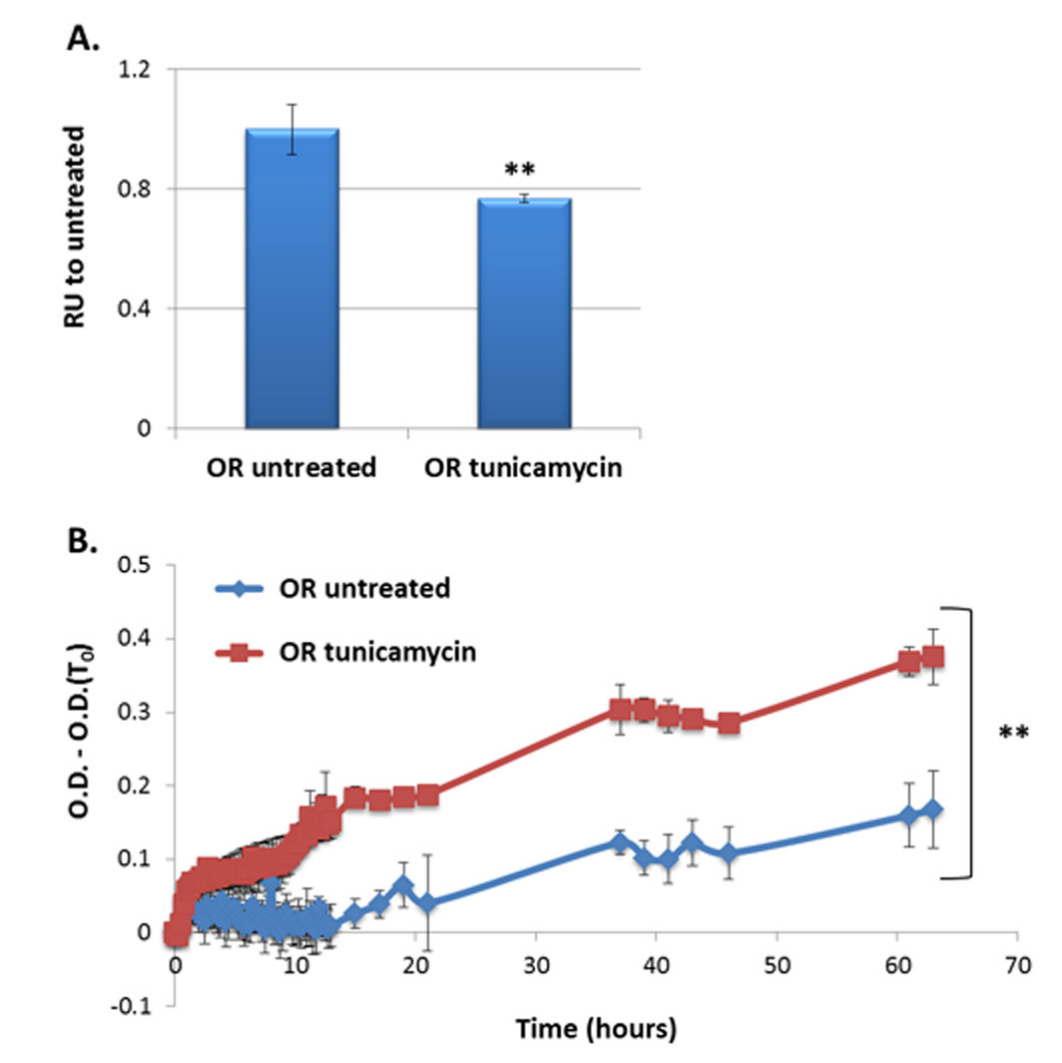
Global proteome turbidity of flies treated with tunicamycin. **A.** Validation of inhibition of glycosylation by tunicamycin using periodic acid-Schiff which stains protein glycans, in treated versus untreated flies. **B.** Turbidity levels of total soluble protein extracts (1 mg/ml) from flies reared on culture medium lacking or containing tunicamycin. All optical densities were measured at 340 nm and their first measurement (O.D. (T_0_)) was subtracted in order to eliminate background absorbance of the samples. Appropriate number of biological (*n* = 4) and technical (*n* = 3) replicates were conducted for each experiment. **P < 0.01.

To examine the effect of ER stress on global accumulation of misfolded/unfolded proteins we used tunicamycin. Tunicamycin is an inhibitor of the UDP-N-acetylglucosamine-dolichol phosphate N-acetylglucosamine-1-phosphate transferase (GPT), thus blocks the initial step of glycoprotein biosynthesis in the ER [31]. As a result, misfolded glycoproteins accumulate in the ER, leading to ER stress [31]. Consequently the misfolded proteins are translocated to the cytosol were they are degraded by the ubiquitin-proteasome machinery (ER-associated degradation - ERAD). We postulated that accumulation of misfolded and unfolded proteins in the ER lumen will lead to higher global aggregation. Wild type (Oregon R, OR) Drosophila were fed on either tunicamycin-containing medium or on regular medium throughout their lifetime and total proteins were extracted using a standard protein extraction procedure. Insoluble components, likely comprising preexisting protein aggregates, membrane particles and other cell debris were removed by centrifugation. The soluble fraction was subjected to periodic acid-Schiff staining, which detects protein glycans, showed that tunicamycin treated flies had lower level of soluble glycoproteins as expected due to the inhibition glycosylation by the drug (**Fig. 1A**). Turbidity analysis of the total proteome in the soluble fraction indicated a modest increase of turbidity over time in the samples from the untreated control flies, reflecting a basal propensity for aggregation. In contrast, turbidity of extracts from tunicamycin treated flies was markedly higher with a significant difference between the two samples noticeable already 9.5 h after the beginning of measuring turbidity (**Fig. 1B**). Thus, the turbidity assay provides a quick and easy readout for the aggregation propensity of the global cellular proteome.

### Expression of aggregation-prone proteins enhances global proteome turbidity

The proteostasis paradigm predicts that over-expression of specific, aggregative protein may cause ER stress leading to aggregation of other aggregation-prone proteins [2]. This should be detectable by monitoring global soluble protein turbidity. To that end, we examined the effect on global protein turbidity of three different aggregative human proteins overexpressed in Drosophila: Tau, Aβ and pVHL.

Tau is an aggregative amyloidogenic protein, and is a hallmark of AD. Expression of human Tau was targeted to the fly eyes *via* the UAS-Gal4 system using the GMR-Gal4 driver [32].

**Figure 2.**
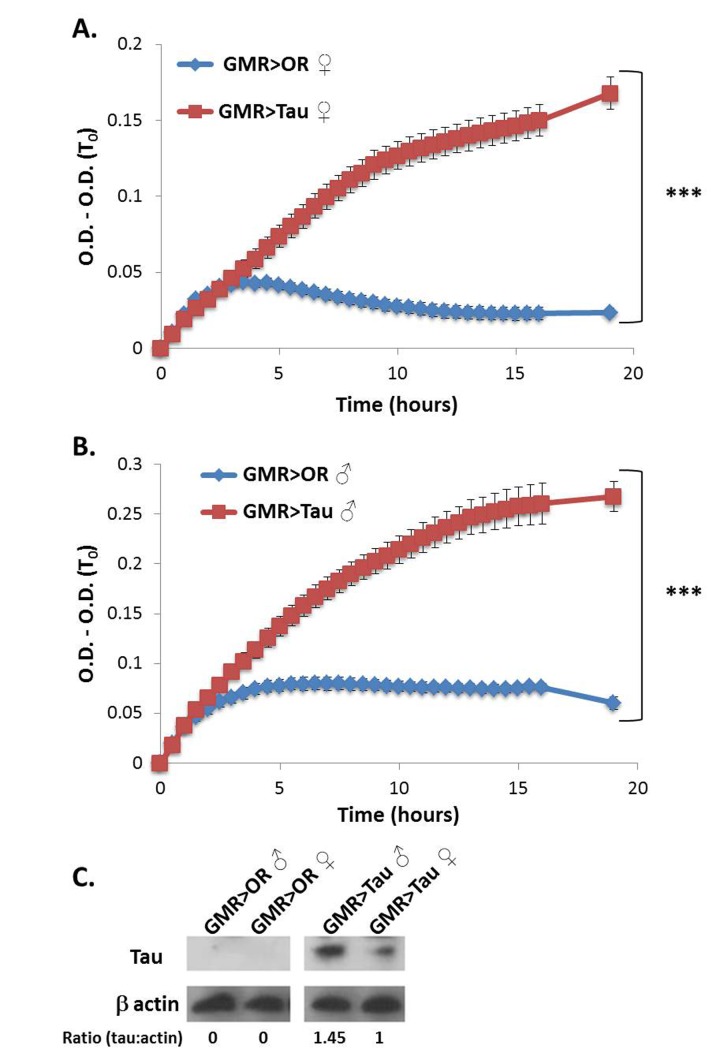
Global proteome turbidity of flies over expressing Tau protein *via* GMR-Gal4. **A** and **B.** Turbidity levels of the soluble fraction of total protein extracts (1 mg/ml) from flies over expressing Tau protein in females (**A**) versus males (**B**). All optical densities were measured at 340 nm and their first measurement (O.D.(T0)) was subtracted in order to eliminate background absorbance of the samples. ♀ = females, ♂ = males. **C.** Western blot analysis showing the levels Tau protein in whole protein extracts from flies heads. β actin was used as loading control. The ratio between Tau protein and β actin is indicated. Appropriate number of biological (*n* = 5) and technical (*n* = 3) replicates were conducted for each experiment. ***P < 0.001.

Global proteome turbidity of the soluble fraction from fly head extracts was found to be significantly higher in samples from flies expressing human Tau compared to control flies which carry GMR-Gal4 only (**Fig. 2A** and **2B**). This was true for both sexes, yet males displayed higher values than females. This is expected since the GMR-Gal4 driver resides on the X chromosome which is expressed in male files considerably higher than in females [33] and is evident as 1.45 higher level of the Tau protein in Tau expressing males than in females (**Fig. 2C**).

**Figure 3.**
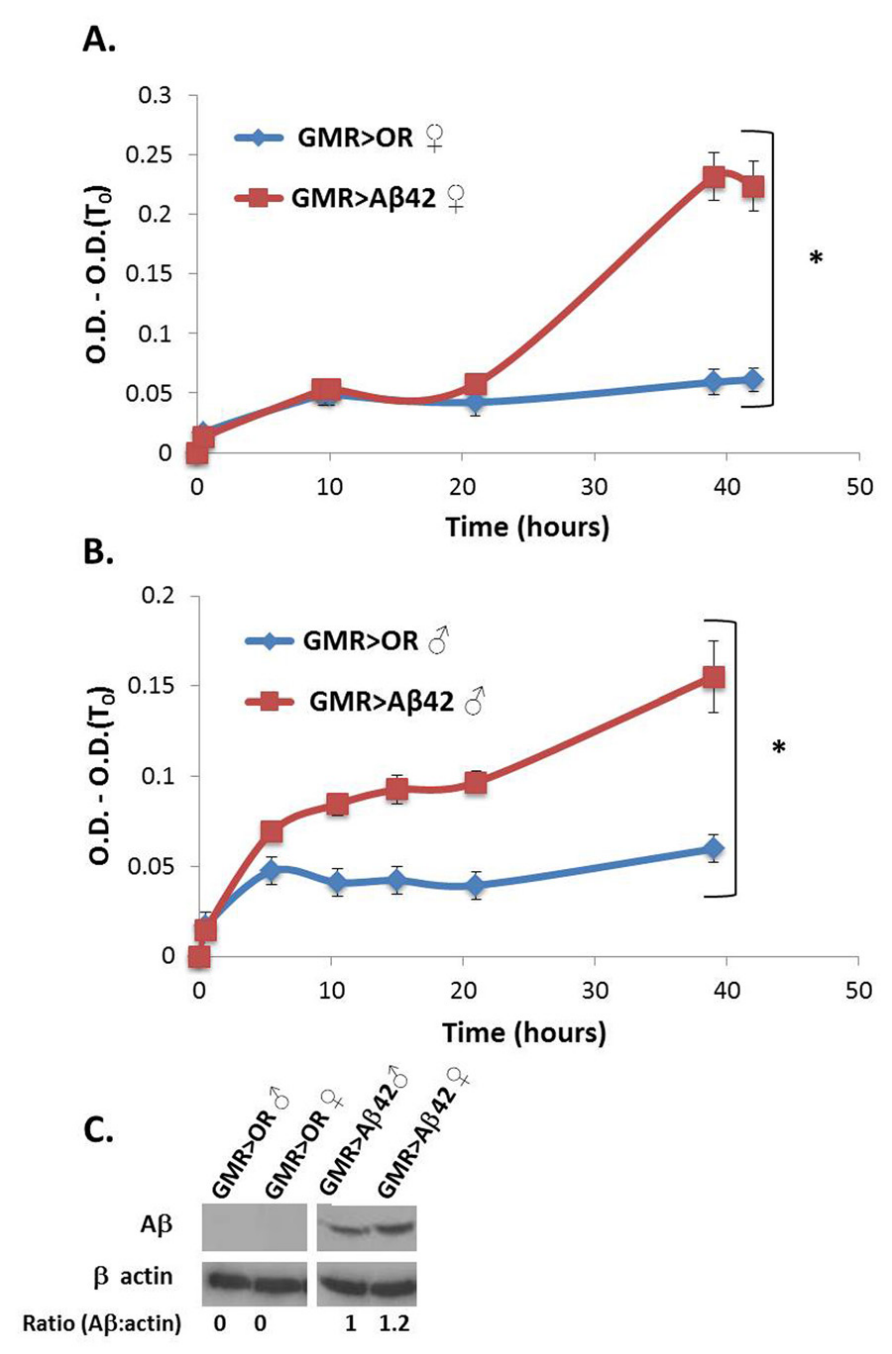
Global proteome turbidity of flies over expressing Amyloid-b protein *via* GMR-Gal4. **A** and **B.** Turbidity levels of soluble protein extracts (1 mg/ml) from flies over expressing Aβ protein females (**A**) versus males (**B**). All optical densities were measured at 340 nm and their first measurement (O.D.(T_0_)) was subtracted in order to eliminate background absorbance of the samples. ♀ = females, ♂ = males. **C.** Western blot analysis showing the levels Aβ protein in whole protein extract from flies heads. β actin was used as loading control. The ratio between Aβ protein and β actin is indicated. Appropriate number of biological (*n* = 3) and technical (*n* = 3) replicates were conducted for each experiment. *P < 0.05.

Over expression of the Alzheimer’s-associated Aβ polypeptide in Drosophila eyes also resulted in significantly enhanced global proteome turbidity of the soluble fraction from head protein extracts compared to samples from control flies carrying GMR-Gal4 only (**Fig. 3**). This was true for both sexes. The higher turbidity observed in females is because they carry two copies (homozygous) of the GMR-Gal4 driver used whereas males carry only one copy. This is reflected in higher expression of the Aβ protein (1.2 fold higher) in the tested females (**Fig. 3C**).

**Figure 4.**
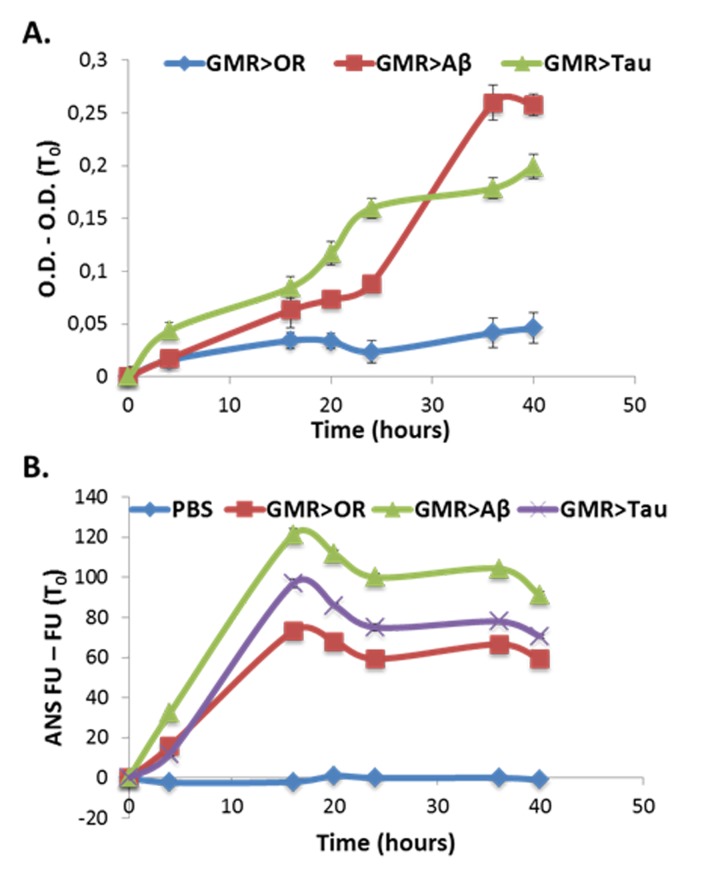
Global proteome turbidity of soluble extracts from flies over expressing Aβ or human TAU by GMR-Gal4. **A.** Turbidity levels of soluble protein extracts (1 mg/ml) from flies over expressing Aβ or Tau proteins. All optical densities were measured at 340 nm and their first measurement (O.D. (T0)) was subtracted in order to eliminate background absorbance of the samples. **B.** ANS fluorescence analysis of soluble protein extracts (1 mg/ml) from flies over expressing Aβ or Tau proteins. Note that errors bar is very small and therefore invisible. Appropriate number of biological (*n* = 3) and technical (*n* = 3) replicates were conducted for each experiment.

We wished to obtain an independent measure of protein aggregation in these experiments. To that end, in parallel to the turbidity assay, we employed a fluorophore. Fluorophores, *e.g.*, Thioflavin-T, are routinely used for evaluating protein aggregation. Here we used ANS commonly employed for monitoring hydrophobic aggregation. The fluorescent properties of ANS change as it binds to hydrophobic regions on the protein surface [34]. **Figure 4** shows that ANS fluorescence can detect differences in propensity for aggregation between the experimental and control samples (expressing *vs*. non expressing the aggregative proteins examined, Aβ or Tau, respectively). Note that there is some aggregation in the control sample (expressing only GMR-Gal4) but this GMR-Gal4 exists also in the experimental samples (which also express Aβ or Tau), underscoring the fact that this measurement is relative and should always be done with the appropriate control sample. Interestingly, as **Figure 4** indicates, measuring global turbidity is more sensitive than ANS fluorescence of the samples. The change in turbidity is primarily due to aggregation of proteins since prior treatment of the samples with either proteinase K or trypsin reduces turbidity markedly (**Fig. S2**).

We next examined global protein turbidity following expression in flies of another aggregative protein, namely the wild type human pVHL and its three mutant versions. pVHL is a tumor suppressor protein whose loss of function is associated with the VHL cancer syndrome [35]. Wild type pVHL has a molten globule conformation and is prone to aggregation [36]. We previously showed that various mutations in pVHL differentially enhance its aggregation propensity *in vitro* [6]. For example, N78S and F136L are structural mutations which alter the folding of pVHL, and leading to higher aggregation propensity than wild type pVHL. Y98H has only a slight effect on pVHL folding and aggregation, yet directly affects its HIF1-a binding site, hence displays severe loss-of-function [6].

**Figure 5.**
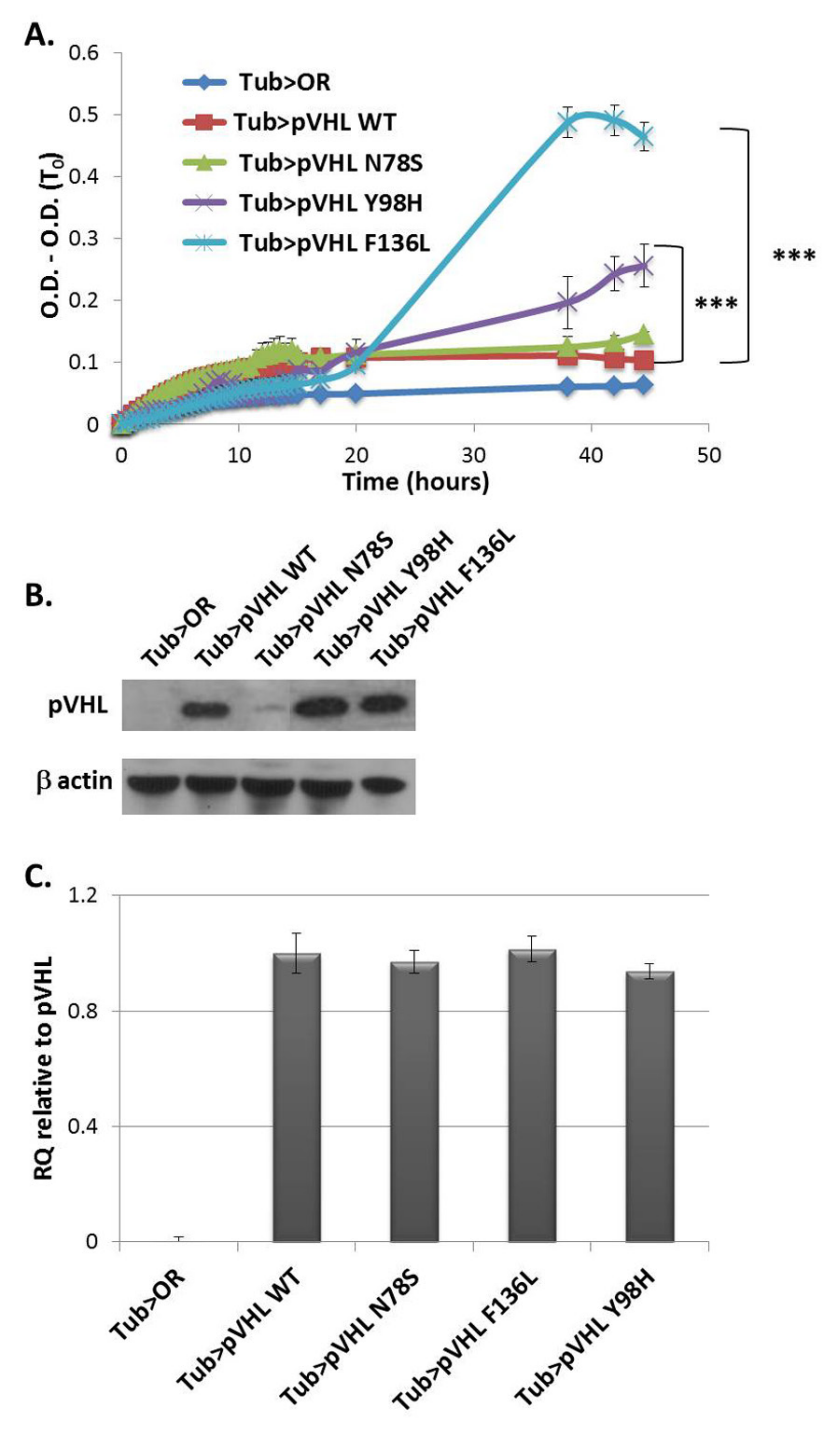
Global proteome turbidity of soluble fraction from flies over expressing human VHL protein by tubulin Gal4. **A.** Turbidity levels of total protein soluble extracts (1 mg/ml) from flies expressing the wild type VHL protein or its mutant versions. All optical densities were measured at 340 nm and their first measurement (O.D.(T_0_)) was subtracted in order to eliminate background absorbance of the samples. **B.** Western blot analysis showing the levels of VHL protein in whole soluble protein extract from flies. β actin was used as loading control. **C.** Real-time qPCR analysis of pVHL transcript in the flies over expressing human VHL protein *via* tub-Gal4. Expression of each gene was normalized to rp49 gene which was used as an internal control. Bars represent SEM (*n* = 5). Appropriate number of biological (*n* = 3) and technical (*n* = 5) replicates were conducted for each experiment. ***P < 0.001.

Wild type pVHL and the three different mutant versions were over-expressed ubiquitously in flies using the tubulin Gal4 driver. Global turbidity of total soluble proteins extracted from whole fly bodies indicated that all four variants of pVHL examined yielded higher turbidity levels than flies from the parental strain expressing only tub-Gal4 (**Fig. 5A**). Turbidity levels of the samples from F136L- and Y98H- expressing flies was 5 and 2.5 fold higher than from flies expressing wild type pVHL, in accordance with their high and moderate propensity to aggregate, respectively [6]. Surprisingly, samples from flies expressing the N78S mutant displayed similar turbidity levels as samples from flies expressing wild type pVHL. This may be due to the markedly low expression level of the N78S protein in the fly soluble fraction (**Fig. 5B**). Real time qPCR analysis indicated that all pVHL transgenes used had similar mRNA levels (**Fig. 5C**). Thus, the N78S mutant protein, which was shown to have short half-life [37], is rapidly degraded thus contributing less to proteome turbidity.

### Induced ER stress in cultured cells results in elevated global proteome turbidity

**Figure 6.**
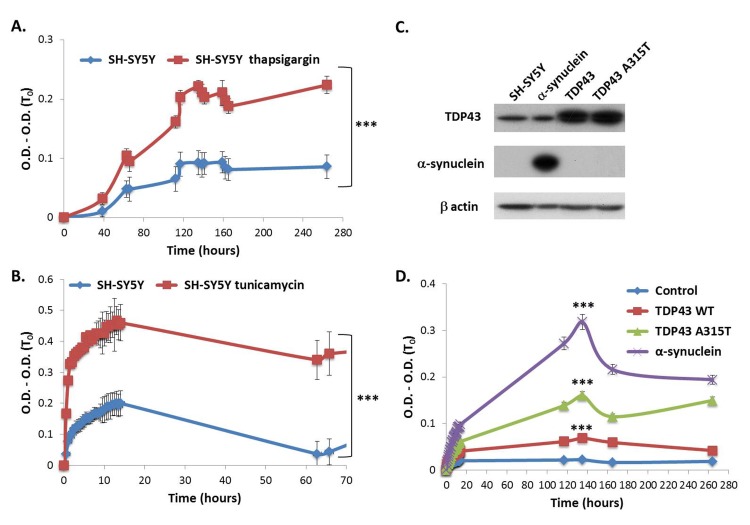
Global proteome turbidity of neuronal cells (SH-SY5Y). **A.** Turbidity levels of total soluble protein extracts (1 mg/ml) from neuronal cells (SH-SY5Y) treated with thapsigargin or untreated cells as a control. **B.** Turbidity levels of total soluble protein extracts (1 mg/ml) from neuronal cells (SH-SY5Y) treated with tunicamycin or untreated as a control. **C.** Western blot analysis showing the levels of α-synuclein, TDP43 or its mutant version TDP43 A315T protein in whole protein extract from SH-SY5Y cells. β actin was used as loading control. **D.** Turbidity levels of total soluble protein extracts (1 mg/ml) from neuronal cells (SH-SY5Y) stably expressing TDP43 or its mutant version TDP43 A315T or α-synuclein. All optical densities were measured at 340 nm and their first measurement (O.D. (T_0_)) was subtracted in order to eliminate background absorbance of the samples. Appropriate number of biological (*n* = 3) and technical (*n* = 3) replicates were conducted for each experiment. ***P < 0.001.

To examine whether the results obtained in the fly model could be recapitulated also in human cells in culture, we treated neuroblastoma cells (SH-SY5Y) with tunicamycin or thapsigargin, both of which cause ER stress. Turbidity levels of soluble total protein extracts from cells treated with either drug were significantly higher (2 fold) than in untreated cells (**Fig. 6A** and **6B**), in accord with the results from flies.

We next examined the effect of over-expression of an aggregative protein in cultured cells on the propensity of the total soluble proteome to aggregate, using the turbidity assay. To that end we produced neuronal cells (SH-SY5Y) stably expressing α-synuclein or TDP43 or (**Fig. 6C**). a-synuclein is associated with Parkinson’s disease and the TAR DNA-binding protein 43 (TDP43) is involved in amyotrophic lateral sclerosis (ALS) and frontotemporal dementia (FTLD-TDP) [38,39]. Significantly higher global turbidity levels (> 12 fold) were found in the soluble fraction from cells expressing a-synuclein, in comparison to naive SH-SY5Y cells (**Fig. 6D**). For TDP43 we expressed two versions—the wild type TDP43 and a mutant A315T version, associated with rare hereditary form of ALS known to have a more severe neurotoxic phenotype, which has higher propensity for aggregation than the wild type counterpart [40]. Indeed, cells expressing A315T exhibited ~2.5 fold higher level of turbidity than cells expressing wild type TDP43 protein and ~10 fold higher than naïve SH-SY5Y cells (**Fig. 6D**).

### Global protein turbidity is increased in brains of Alzheimer’s disease patients

**Figure 7.**
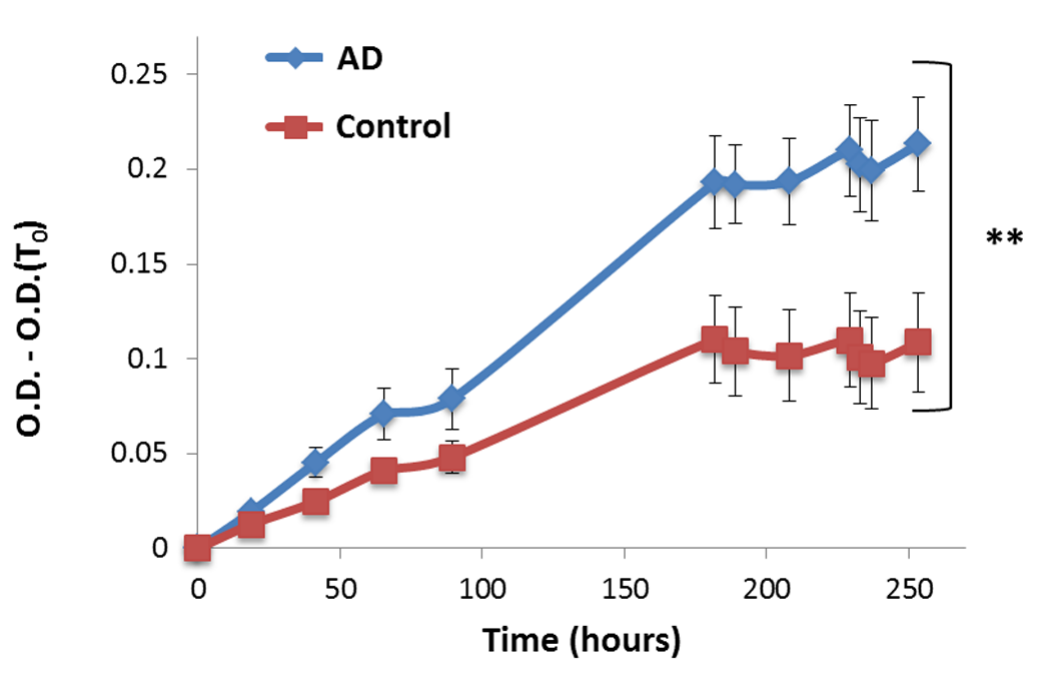
Global proteome turbidity is increased in brains of Alzheimer’s disease patients. Turbidity levels of total soluble protein extracts from 10 brains (5 AD patients, 5 controls). All optical densities were measured at 340 nm wavelength and subtracted with their first measurement (O.D.(T_0_)) in order to eliminate background absorbance of the samples. Appropriate number of biological (*n* = 5) and technical (*n* = 3) replicates were conducted for each experiment. **P < 0.01.

The brain of AD patients displays characteristic aggregates composed mainly of Aβ or Tau proteins. We examined whether global protein turbidity is altered in the soluble fraction of extracts from postmortem AD patient brains (**Fig. S3** shows a western blot of the clear supernatant and the pellet fractions from three independently prepared human brain tissue extracts, demonstrating that the supernatant contains mainly the soluble fraction). **Figure 7** shows that extracts of frontal cortex from AD patients displayed significantly higher (2-fold) turbidity level than samples from brains of healthy age matched controls demonstrating that this assay can easily and quantitatively detect symptoms of protein misfolded diseases.

We expect other protein misfolding diseases (*e.g.*, Parkinson’s disease and Huntington’s disease, type 2 diabetes, some forms of atherosclerosis and various types of cancer [41]) to display changes in total proteome turbidity as revealed for AD patients by the assay described here. It will be interesting, in the future, to identify the proteins composing the aggregates that cause the enhanced turbidity, which may have been recruited by the nucleating aggregative protein. Total proteome turbidity can serve as a diagnostic tool for monitoring progression of protein misfolding diseases. When combined with the identification of the aggregated proteins it could serve as a novel biomarker. This should be particular feasible using body fluids in which the aggregates accumulate such as cerebrospinal fluid and blood for example in neurodegenerative diseases and serum amyloidosis.

In conclusion, we present a facile and practical method for monitoring aggregation propensity in the natural cell environment, by applying the turbidity assay to total protein extracts. It is important to bear in mind that this measurement is relative.

Collectively the results described above, indicate that the propensity to aggregate of the global soluble proteome can be detected under various physiological conditions even in the absence of an apparent stress or aggregation seed as evident from the results of all of the controls used (untreated samples and naïve cells). It probably reflects the relative extent at which the cellular milieu is prone and conducive to protein aggregation. Turbidity levels and kinetics are therefore relative and should be monitored only in comparison to an appropriate control. Furthermore, standard protein extraction protocols are often specific for a given tissue or organism, precluding comparison of absolute turbidity levels between samples from distinct sources. We observed that nucleation by individual aggregative proteins may enhance global protein aggregation. This implies that under various stresses, which often occur in various diseases, aggregates of the disease-related proteins may function as a seed and recruit other proteins thus enhancing global proteome aggregation.

## Supplementary Material

Supplementary information **Figure S1**. Spectra of global proteome absorbance of soluble fraction from cells or flies.
**Figure S2**. Global proteome turbidity of wild type flies.
**Figure S3**. Western blot of the clear supernatant and the pellet fractions from three independently prepared human brain tissue extracts.
Supplementary information of this article can be found online at
http://www.jbmethods.org/jbm/rt/suppFiles/148.

## Figures and Tables

**Table 1.** Genotypes of flies used for protein extraction.

Protein over-expression	Females	Males
hTAU	yw,P{GMR-Gal4, w+}/FM7a; P{UAS-tau(wt)-1.13,w+}/CyO	yw,P{GMR-Gal4, w+}; P{UAS-tau(wt)-1.13,w+}/CyO
hAβ	yw,P{GMR-Gal4, w+}/yw,P{GMR-Gal4, w+}; P{UAS-APP.Aβ42.B}m26a/CyO [24]	yw,P{GMR-Gal4, w+}; P{UAS-APP.Aβ42.B}m26a/CyO [24]
Control	yw,P{GMR-Gal4, w+}/+; +/+	yw,P{GMR-Gal4, w+}; +/+

**Table 2.** Human brain tissue of Alzheimer’s disease and control cases used in this study.

Case #	Gender	Age at death (yr)	Braak stage	Brain bankidentifier #
Con 1	F	82	0	06/19
Con 2	F	79	1	06/08
Con 3	F	85	0	07/07
Con 4	F	82	1	06/12
Con 5	M	86	0	07/06
AD 1	M	45	5–6	92/47
AD 2	M	44	5–6	92/20
AD 3	F	53	5–6	91/76
AD 4	F	86	6	03/19
AD 5	M	74	6	97/24
